# Levels of autonomy in FDA-cleared surgical robots: a systematic review

**DOI:** 10.1038/s41746-024-01102-y

**Published:** 2024-04-26

**Authors:** Audrey Lee, Turner S. Baker, Joshua B. Bederson, Benjamin I. Rapoport

**Affiliations:** 1 Department of Neurosurgery, Icahn School of Medicine at Mount Sinai, New York, New York USA; 2 Sinai BioDesign, Icahn School of Medicine at Mount Sinai, New York, New York USA; 3 Department of Population Health Science and Policy, Icahn School of Medicine at Mount Sinai, New York, New York USA

**Keywords:** Health care, Biotechnology

## Abstract

The integration of robotics in surgery has increased over the past decade, and advances in the autonomous capabilities of surgical robots have paralleled that of assistive and industrial robots. However, classification and regulatory frameworks have not kept pace with the increasing autonomy of surgical robots. There is a need to modernize our classification to understand technological trends and prepare to regulate and streamline surgical practice around these robotic systems. We present a systematic review of all surgical robots cleared by the United States Food and Drug Administration (FDA) from 2015 to 2023, utilizing a classification system that we call Levels of Autonomy in Surgical Robotics (LASR) to categorize each robot’s decision-making and action-taking abilities from Level 1 (Robot Assistance) to Level 5 (Full Autonomy). We searched the 510(k), De Novo, and AccessGUDID databases in December 2023 and included all medical devices fitting our definition of a surgical robot. 37,981 records were screened to identify 49 surgical robots. Most surgical robots were at Level 1 (86%) and some reached Level 3 (Conditional Autonomy) (6%). 2 surgical robots were recognized by the FDA to have machine learning-enabled capabilities, while more were reported to have these capabilities in their marketing materials. Most surgical robots were introduced via the 510(k) pathway, but a growing number via the De Novo pathway. This review highlights trends toward greater autonomy in surgical robotics. Implementing regulatory frameworks that acknowledge varying levels of autonomy in surgical robots may help ensure their safe and effective integration into surgical practice.

## Introduction

When the first surgical robots entered medical practice, they were truly robotically-assisted systems with no independent decision-making and action-taking abilities^1^. Yet, in the popular imagination, there has always been an anticipated future with autonomous systems performing complex procedures with minimal surgeon intervention. Today, advancements in automation and the growth of artificial intelligence and machine learning have brought this imagined future closer to reality. Adjacent fields such as assistive robotics and industrial robotics are already seeing examples of robots with increasing autonomy that can work with and around humans. While the same degree of autonomy is not yet available in surgery, it is a perceived inevitability that requires careful planning.

The surgical robotics field has changed significantly since the *Automated Endoscopic System for Optimal Positioning* (AESOP) became the first FDA-cleared surgical robot in 1993. The FDA cleared AESOP through the 510(k) Premarket Notification pathway as a Class II (moderate risk) device, which set a precedent for the regulatory evaluation of surgical robots^1^. Since then, surgical robots have progressed from minor supporting roles to more complex autonomous systems. For instance, robots in current surgical practice range from leader-follower systems like the *da Vinci Surgical System* (Intuitive Surgical, USA), where the robot does not perform tasks automatically but is entirely controlled by the surgeon, to systems like the *TSolution One* (Think Surgical, USA), where the robot generates patient-specific operative plans and automatically performs bone milling while the surgeon watches. However, the taxonomic tools to describe and regulate these robotic systems have remained static and narrow.

The prevailing classification of surgical robots utilizes organizing frameworks and definitions that carry legacy constructs from industrial robotics and autonomous motor vehicles and do not do justice to surgical robotics today. We provide commonly used definitions and standards for machinery used in surgery in Supplementary Table 1^2–13^. For example, the IEC/TR 60601-4-1 Medical Electrical Equipment Technical Report defines a taxonomy for degrees of autonomy in medical robotics. However, it focuses solely on technical metrics while failing to discuss practical developmental benchmarks and human-robot interactions critical for ensuring patient and surgeon safety as the field advances. Since 2015, the FDA has advocated for the term “robotically-assisted surgical devices” instead of “surgical robots” to emphasize that all cleared systems have no robotic autonomy, as they require the surgeon’s direct and continuous control to move and activate surgical instruments^10–12^. By this definition, the surgeon is entirely responsible for the safety of the procedure and is expected to maintain proper training across different models of robotically-assisted surgical devices^2,12^.

The growing integration of automation and machine learning into patient-specific surgical planning and task execution now challenges the presumption that surgical robots lack autonomy. This development also makes it increasingly difficult to regulate and streamline surgical workflows around such technologies. The lack of adequate tools to capture these trends toward increasing robotic autonomy complicates the roles and responsibilities of surgeons and manufacturers and raises many potential legal and ethical considerations. For instance, questions such as who is legally responsible for procedural safety as surgical tasks are increasingly automated, what additional technical competencies would surgeon training programs require, and who ensures that the machine learning models in these systems continue to perform adequately over time highlight the need for further clarity on regulatory paradigms. For surgical robots to live up to their technological potential, standards organizations, regulatory agencies, and medical societies need a unifying framework tailored to surgical robotics. Such a framework would enable the development of regulatory standards and surgical practice parameters to provide reasonable assurance of the safety and effectiveness of modern surgical robots.

We analyzed all Class II-risked surgical robots cleared by the FDA since 2015 through the lens of a Levels of Autonomy in Surgical Robotics (LASR) taxonomy to identify trends in the regulatory process for surgical robotics and automation. This review is intended to highlight key considerations for the development of regulation and surgical practice parameters around increasingly autonomous robotic systems.

## Results

**Fig. 1:**
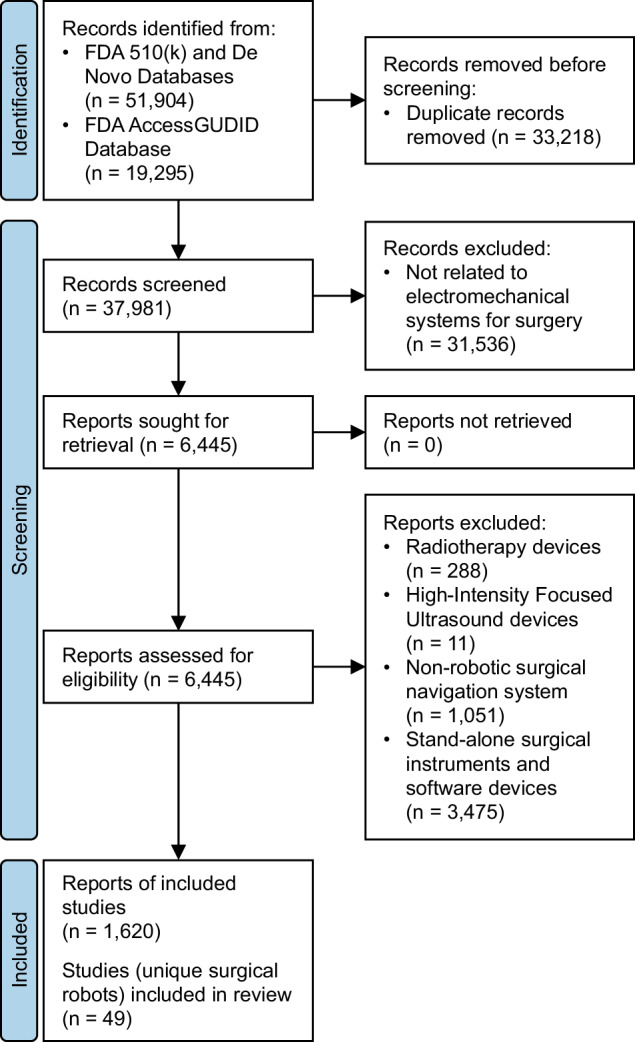
PRISMA flow diagram for identifying FDA-cleared surgical robotic devices. Study selection process.

**Fig. 2:**
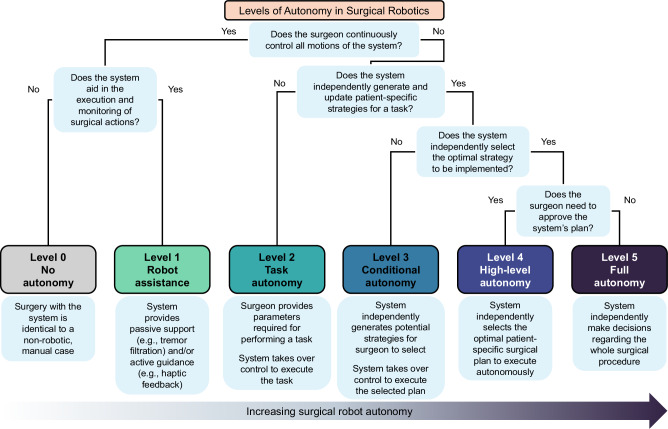
Levels of Autonomy in Surgical Robotics (LASR) taxonomy. Characteristics of each level of autonomy.

After duplicate removal, we manually screened 37,981 database records, from which we reviewed 6445 full-text reports. 1620 reports were grouped to identify unique surgical robots (Fig. 1). Each surgical robot was then classified using the LASR scale (Fig. 2).

**Fig. 3:**
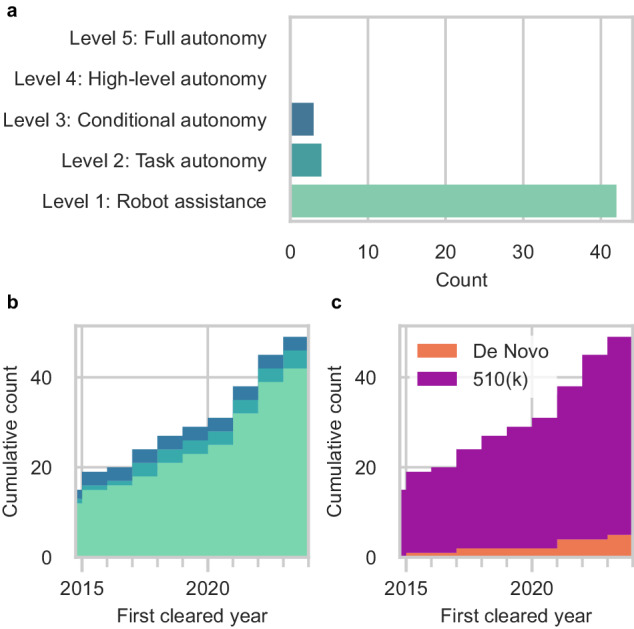
Number of FDA-cleared surgical robots, classified by their level of autonomy. **a** Current total surgical robots. **b** Cumulative count of new surgical robots over time by the year of first FDA clearance. **c** Cumulative count of FDA regulatory pathway taken by new surgical robots over time by the year of first FDA clearance.

We identified 49 unique surgical robots with our search strategy. We considered most robotic systems as Level 1 (Robot Assistance) (42 systems [86%]) which operate under continuous surgeon control (Fig. 3a). We also considered 4 systems (8%) as Level 2 (Task Autonomy) surgical robots that could execute preprogrammed, automated actions for a specific surgical task. The most advanced surgical robots cleared by the FDA reached Level 3 (Conditional Autonomy) (3 systems [6%]), which could generate patient-specific strategies for a surgical procedure. There were no examples of Level 4 and Level 5 surgical robots.

After the surgical robots were organized by the year of their first FDA clearance, we observed that 15 robotic systems were first cleared prior to 2015 but obtained addendum clearances for their expanded capabilities within our search period (Fig. 3b). 34 entirely new surgical robots were introduced for Level 1 through Level 3 since 2015. Since 2017, there has been a gradual shift towards increased task automation with the introduction of new Level 2 surgical robots. Only one additional Level 3 surgical robot was cleared by the FDA in 2015. Most of the surgical robots were cleared through the FDA’s 510(k) pathway (44 systems [90%]). A smaller but growing number of systems were introduced through the De Novo pathway (5 systems [10%]) (Fig. 3c). Only 19 of the surgical robots (39%) had accompanying clinical testing data. These included all 3 of the Level 3 surgical robots (100%), 3 of the Level 2 surgical robots (75%), and 13 of the Level 1 systems (31%). 2 of the surgical robots were reported to have machine learning-enabled software features in their submissions to the FDA. However, 3 additional surgical robots were marketed to have machine learning-enabled capabilities on their product websites that were not in their FDA summary documents.

**Fig. 4:**
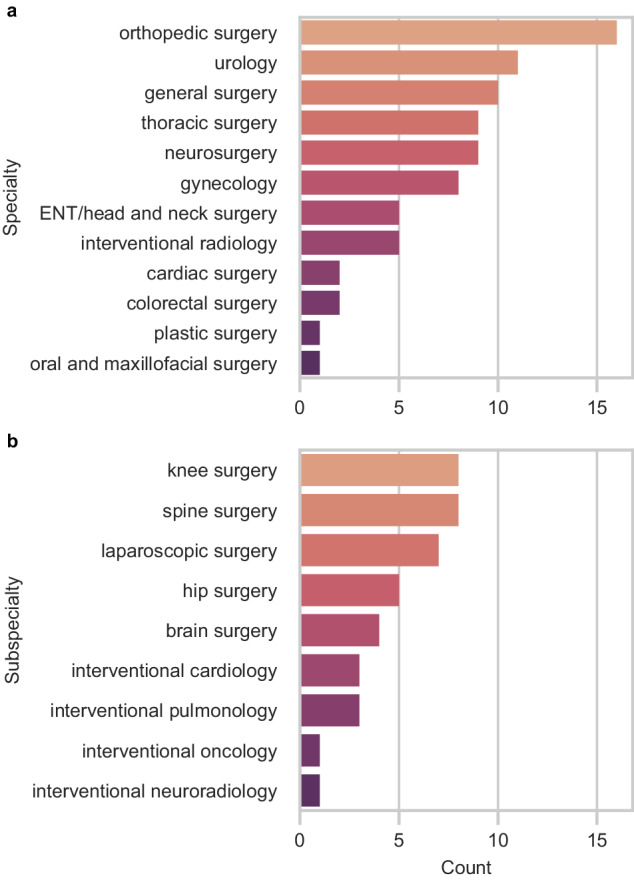
Number of FDA-cleared surgical robots by intended specialty and subspecialty. **a** By specialty. **b** By subspecialty.

Nearly all robotic systems were designed to accommodate a variety of procedures across different specialties (73%) (Fig. 4a). Orthopedic surgery was the fastest-growing specialty in surgical robotics, with 33% of all new robotic systems introduced since 2015 intended for spine, knee, and hip surgeries (Fig. 4b). The number of surgical robots for urology also expanded with 11 new or improved robotic systems, followed by general surgery (10 systems), thoracic surgery (9 systems), and neurosurgery (9 systems). There were fewer robotic systems for other specialties, including otolaryngology (ENT)/head and neck surgery, interventional radiology, and plastic surgery.

**Fig. 5:**
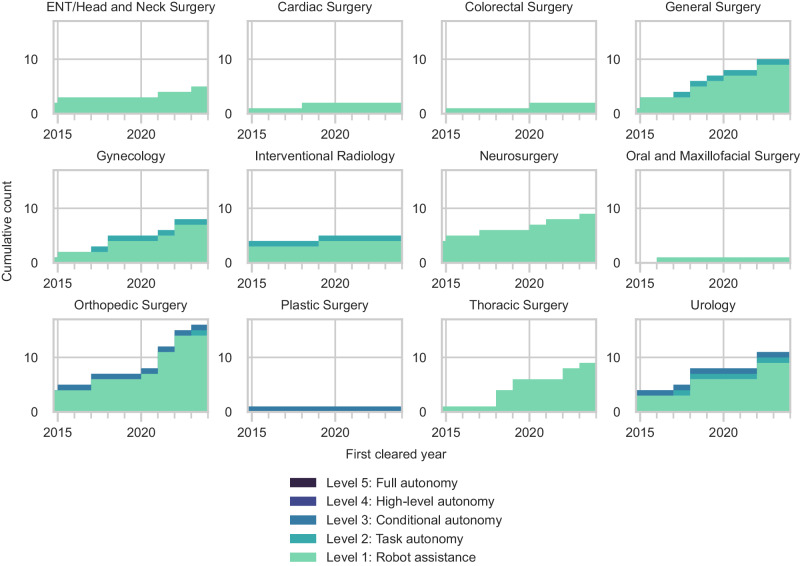
Cumulative count of new surgical robots over time by the year of first FDA clearance. Organized by surgical specialty.

Most specialties have only Level 1 surgical robots (Fig. 5). The three most advanced Level 3 robotic systems were intended for autonomously generating and executing patient-specific plans for bone milling in orthopedic surgery, prostate biopsy in urology, and hair follicle extraction in plastic surgery. Orthopedic surgery, urology, general surgery, gynecology, and interventional radiology were the only specialties with Level 2 surgical robots.

An abbreviated summary of the Level 2 and Level 3 robotic systems is provided in Table 1. All surgical robots and their data collected in this study are presented in Supplementary Data 1.

**Table 1:** Level 2 and level 3 surgical robots

Company Name	Device Name	Level of Autonomy	Key Features	Specialty/Subspecialty	Pathway	First FDA Clearance Year	AI/ML Enabled? (FDA/Marketed)
Asensus Surgical	Senhance	2	“Augmented intelligence” for “real-time visual guidance” to “automatically [identify] structures of anatomy, safety hazards and more.” Haptic sensing and feedback. Automated camera control and eye tracking camera control. Digital fulcrum point minimizes torque at incision site. Open platform architecture.	general surgery, gynecology, laparoscopic surgery	510(k)	2017	yes/yes
Corindus, Siemens Healthineers	CorPath GRX	2	Set of automated movements to aid in navigation and manipulation. Controlled by joystick controller and console touch screen at remote workspace.	interventional radiology, interventional cardiology	510(k)	2012	no/no
Procept Biorobotics	AquaBeam Robotic System	2	Combined cystoscopic visualization with ultrasound imaging enable personalized treatment planning. Once the treatment map is complete, prostate tissue is removed using a robotically controlled, heat-free waterjet.	urology	De Novo	2017	no/no
Think Surgical	TMINI	2	Following a CT-based 3D surgical plan, the robotically controlled handheld tool “automatically compensates for surgeon hand movement.”	orthopedic surgery, knee surgery	510(k)	2023	no/no
Biobot	iSR’obot Mona Lisa 2.0	3	A targeted biopsy plan is proposed by the system and can be customized. A virtual pivot point ensures multiple needle entries through the same channel. “The robotic needle guide and needle stopper are automatically positioned.”	urology	510(k)	2011	no/no
Think Surgical	TSolution One	3	Planning software designs a procedure tailored to the patient’s unique anatomy. Active robot automatically prepares the bone while the surgeon supervises the procedure by watching the monitor and cutting tool to ensure proper system operation.	orthopedic surgery, knee surgery, hip surgery	510(k)	2015	no/no
Venus Concept	ARTAS iX System	3	“Intelligent hair transplant platform to utilize state-of-the-art robotic and artificial intelligence technology.” “Artificial intelligence consistently analyzes, monitors, and tracks each hair follicle’s characteristics and adjusts for patient movement. Machine learning recognizes and digitally maps past, current, and future grafts, and learns new patterns to deliver continuous improvement.” Image-guided robotic alignment technology to harvest each follicular unit at the optimal angle.	plastic surgery	510(k)	2011	no/yes

## Discussion

Since 2015, the FDA has cleared nearly 50 surgical robots, including new systems and existing systems with expanded capabilities. Research developments at the intersection of automation, machine learning, and robotics continue to advance the levels of autonomy embodied by surgical robots and challenge traditional paradigms. However, the current frameworks for classifying and regulating these robotic systems have not kept pace with these advancements, as they emphasize only technical capabilities and are not specific to surgical robots. There is a need for a clear framework that captures the roles of surgeons during procedures with these systems while allowing room for growth as the field evolves. Such a framework would help to establish a common baseline to develop regulatory standards and practice parameters that promote procedural safety and liability management.

We utilized a Levels of Autonomy in Surgical Robotics (LASR) classification system to capture the technological and regulatory trends in surgical robotics involving automation and robotic autonomy. By categorizing robotic systems with LASR, we can appreciate that the field has progressed with features of higher levels of robotic autonomy, like patient-specific surgical plan generation and task automation. We observed that the most advanced surgical robots cleared for clinical use reached Level 3 capabilities. LASR may help guide the development of more focused regulatory standards and practice parameters for surgical robotics.

The FDA currently regulates all surgical robots as Class II (moderate risk) devices, which follows the precedent established by the clearance of prior Level 1 robotic systems^11^. The FDA considered most surgical robots through the 510(k) Premarket Notification pathway, which requires a demonstration of substantial equivalence to a legally marketed device or “predicate” through non-clinical testing^14^. Five robotic systems—the *AquaBeam Robotic System* (Procept BioRobotics, USA), *Anovo Surgical System* (Momentis Surgical, Israel), *MARS Surgical System* (Levita Magnetics, USA), *Iotasoft Insertion System* (iotaMotion, USA), *Galen ES* (Galen Robotics, USA)—were introduced via the De Novo pathway. Devices progressing through the De Novo pathway do not have predicates and instead undergo a risk-based classification. If the FDA grants the De Novo request for a device, the device may serve as a predicate for future iterations to pursue the 510(k) pathway^15^. Current Level 2 and Level 3 surgical robots mitigate risk by requiring the surgeon to review and decide what automated actions or strategies the robotic system should execute. While this approach of placing the burden of responsibility on the surgeon as the ultimate decision-maker may be adequate for the clearance of current surgical robots, it may not be sufficient for future robotic systems with higher levels of autonomy. Practical developmental benchmarks based on LASR are needed to delineate transitions in levels of autonomy and recognize diverse modes of human-robot interactions in surgical robotics. Predicate creep—a repetitive cycle of technology changes between 510(k) clearances that may result in the sudden introduction of devices with high levels of complexity—has been identified in surgical robotics^16,17^. To ensure that future robotic systems with higher levels of autonomy are appropriately introduced with actual clinical evidence of safety, organizing frameworks that expand on LASR may be used to guide the definition of substantial equivalence requirements for more advanced surgical robots. Alternatively, there has been speculation that future Level 4 and Level 5 surgical robots may be deemed Class III (high risk) devices that require Premarket Approval (PMA)^18^. PMA is the most stringent regulatory pathway for medical devices that often involve new concepts not found in existing devices. This pathway may ensure rigorous evaluation of these technologies, thereby increasing confidence in their safety and effectiveness. However, it may simultaneously create bottlenecks in the innovation of Level 4 and Level 5 surgical robots. The regulatory approval of Class III devices often requires significantly more time and cost investment than that of Class II devices^5,14,18^. As advancements in surgical robotics are primarily driven by key players in the market, this can potentially impose barriers to entry for newer and smaller surgical robotics companies. Medical specialties with comparatively smaller device markets may fall further behind in surgical robotics innovation. Moreover, there is concern that surgical robots at these levels may be considered systems that practice medicine because of their independent decision-making capabilities^18^. Since regulating the practice of medicine is more in the realm of medical societies rather than the FDA, these societies need to work alongside regulatory agencies and engineers to determine how to evaluate these surgical robots.

At present, most instances of surgical robot autonomy involve closed-loop control paradigms. However, a growing number of surgical robot manufacturers reported machine learning-enabled software features in their systems. Consistent with the FDA’s publicly available list of artificial intelligence and machine learning-enabled devices^19^, we identified two surgical robots—the *Senhance Surgical System* (Asensus Surgical, USA) and *Cirq Robotics* (Brainlab, Germany)—with reported machine learning-enabled capabilities. While these capabilities are currently limited to aiding in patient registration, automated endoscope camera control, and digital 3D measurements, it is evident that machine learning plays a growing role in advancing robotic autonomy. In addition, we identified three surgical robots—the *Artas iX System* (Venus Concept, Canada), *CORI Surgical System* (Smith and Nephew, USA), *Artemis* (Eigen, USA)—marketed to have artificial intelligence and machine learning-enabled capabilities not mentioned in their FDA summary documents. While it is beyond the scope of this review to understand this discrepancy, it underscores the need for careful consideration of how surgical robot software components, particularly those integrating machine learning, are evaluated along with the system’s hardware components. Machine learning algorithms may propagate biases that exist within training data^20^. When these biases are not rectified, they may lead to unintended consequences. In the context of surgical robots, this could pose a significant risk, especially if these machine learning algorithms are part of automated tasks. Questions such as “Should clinical data be required in these cases?” and “Is it safe to declare purely software medical devices or non-machine learning-enabled surgical robots as predicates?” must be clarified. Hence, machine learning as a means of advancing surgical robot autonomy may need to be scrutinized more, if not the same, as other means of robot automation.

Technological progress in surgical robotics requires the parallel evolution of regulatory frameworks and surgical practice workflows specific to each level of autonomy. Recognizing the different levels of autonomy of surgical robots is essential to their safe and effective integration into surgical practice. Nevertheless, levels of autonomy do not provide an all-encompassing classification of risk, as risk is ultimately procedure-specific. Future work can address these limitations by incorporating surgical context into the concept of levels of autonomy^21^. While the regulation of increasingly autonomous surgical robots is yet to be determined, establishing practice parameters for each level of autonomy will be needed regardless.

Surgical robotics is evolving with new modes of surgeon-robot interactions, the integration of machine learning, and the potential for higher levels of robotic autonomy. How surgical robots are categorized and regulated must keep up with these technological advancements. The Levels of Autonomy in Surgical Robotics scale helped to reflect the modern state of the field with an understanding that increasing robotic autonomy is seemingly inevitable given progress in adjacent fields. Recognizing these trends in regulatory frameworks will be essential to ensuring patient and surgeon safety in this developing area of medical technology.

## Methods

This study followed the Preferred Reporting Items for Systematic Reviews and Meta-Analyses (PRISMA) reporting guidelines^22^.

### Data collection

All records from the FDA 510(k) and De Novo databases with a decision date since January 1, 2015 were downloaded. Duplicate records by 510(k) Number and De Novo Number were automatically removed. AccessGUDID (Global Unique Device Identification Database) records were also collected using the online portal system with search query ((robot*) AND (surg*)) OR ((robot*) AND (intervention*)). No restriction on time was applied to the AccessGUDID search, as it was not a filter option. Duplicate AccessGUDID records by Public Device Record Key were automatically removed. Record collection for all databases occurred on March 2, 2023 and on December 11, 2023 using the same search method.

### Eligibility criteria and screening process

One researcher (AL) independently and manually reviewed the database fields of all records and performed duplicate checking of all records on separate days. We excluded records with Device Classification Names (510(k) and De Novo), and Global Medical Device Nomenclature Terms (AccessGUDID) that were not directly related to electromechanical systems used for surgery. Examples of names and terms that we deemed unrelated were “polymer patient examination glove”, “wheelchair, powered”, and “general surgical procedure kit”. Examples of names and terms that we deemed related included “system, surgical, computer-controlled instrument”, “robotic surgical arm system”, and “robotic surgical navigation system”.

Reports—the full-text summary and statement documents accompanying submissions to the FDA, or device-specific pages on AccessGUDID—were retrieved and manually assessed by one researcher (AL) alongside manufacturer and distributor websites to identify eligible studies or surgical robots. We defined “surgical robot” according to the International Organization for Standardization (ISO) and International Electrotechnical Commission (IEC) definitions in ISO 8373, IEC 80601-2-77, and IEC 60601-4-1 as medical electrical equipment with a degree of autonomy that incorporates a computer-controlled electromechanical component intended to actuate, position, orient, or manipulate a surgical instrument—an invasive device with an applied part that could administer energy or invade into the patient’s body through an incision on the skin or inner surface of a natural orifice^8^. By this definition, we excluded robotic radiation therapy systems and robotic high-intensity focused ultrasound systems which do not require incisions, and surgical navigation systems that lack a computer-controlled electromechanical component. We also excluded stand-alone surgical instruments and software devices that were not intended for use with a surgical robot. Conversely, we included surgical instruments, software, and accessories that were intended for use with a surgical robot.

Since the FDA requires a new 510(k) submission for changes or modifications to existing devices and AccessGUDID documents all versions or models of devices, there were multiple reports corresponding to each unique surgical robot. One review author (AL) manually assessed all eligible reports alongside corresponding manufacturer and distributor websites to identify unique devices (Supplementary Methods). Older-generation surgical robots from the same company were grouped with their newest versions.

### Levels of Autonomy in Surgical Robotics (LASR) taxonomy

In concordance with prior research and standardization efforts, we defined a Levels of Autonomy in Surgical Robotics (LASR) scale that clarifies the division of roles between surgeons and robotic systems during surgery^2,3,5,8,9,18,23,24^. Specifically, we used concepts from the framework on levels of autonomy for medical robotics originally proposed by Yang et al.^18^ and further tailored to surgical robotics by Fosch-Villaronga et al.^23^., Haidegger et al.^5,24^, and Attanasio et al.^2^. based on the emerging ISO and IEC standards^3,8,9^. Building upon these, we introduced additional clarifications informed by surgeon feedback to define the division of roles between surgeons and robots during surgery and human-robot interactions. Thus, the LASR taxonomy classifies each surgical robot by its highest level of autonomy capabilities from 0 (No Autonomy) to 5 (Full Autonomy) (Fig. 2).

#### Level 0—no autonomy

Devices without robotic equipment. The surgeon generates, selects, executes, and monitors all surgical actions, and the device provides no aid in such actions. Surgeries performed with these devices are considered identical to non-robotic manual cases.

By our definition of a “surgical robot”, we excluded Level 0 devices in this review.

#### Level 1—robot assistance

Surgical robots that require the surgeon to control all movements of the system and activation of its surgical instruments directly and continuously. The surgeon generates, selects, executes, and monitors all surgical actions, and the surgical robot aids the surgeon in the execution and monitoring of such actions either with passive support or active guidance. In passive support, the surgeon maintains a free range of motion while the surgical robot provides minor assistance that does not grossly interfere with the surgeon’s intended motion trajectories. Examples include teleoperation, tremor filtration, and tool tracking. In active guidance, the surgical robot provides mechanical support such as haptic feedback or motion constraints to influence the surgeon’s physical actions.

#### Level 2—task autonomy

Surgical robots that can execute and monitor preprogrammed, automated actions for a specific task when selected by the surgeon, without requiring the surgeon’s continuous direct control over the movements and instrument activations. The surgical robot is unable to independently define parameters to generate plans, so the surgeon needs to provide the information required to perform the action. These actions are predictable and designed to reduce variability across procedures. The preprogrammed actions may automate either a few discrete surgical gestures—the smallest meaningful interaction of a surgical instrument with human tissue—or the complete task, which involves a coordinated sequence of multiple surgical gestures^25,26^.

#### Level 3—conditional autonomy

Surgical robots that can propose various patient-specific strategies for surgical tasks or procedures that the surgeon may select from or revise, and then automatically execute and monitor the actions of the surgeon-approved plan. The robotic system extracts parameters from uploaded data streams such as preoperative patient scans to autonomously generate potential strategies for a task, and constantly monitors the surgical environment via methods like real-time intraoperative imaging to update the strategies.

#### Level 4—high-level autonomy

Surgical robots that can generate and proactively select the optimal patient-specific surgical plan and autonomously execute and monitor the plan upon surgeon approval. These robotic systems constantly monitor the surgical environment and autonomously make minor updates to the procedural plan as needed. If extreme changes occur intraoperatively such that the surgical robot’s uncertainty exceeds the limits for guaranteed safety, the robotic system may request the surgeon to safely intervene through methods such as temporarily handing over control to the surgeon or requesting additional inputs. Of note, the surgeon is only required to approve the plan and supervise the procedure; although the surgeon has the option to intervene when they see fit or when requested, the robotic system shall be able to complete the procedure even without surgeon intervention.

#### Level 5—full autonomy

Surgical robots that can independently make decisions regarding the whole surgical procedure, including preoperative workflows. These systems can generate and select the optimal patient-specific surgical plan without prior surgeon approval, and autonomously execute and monitor the plan. Although the surgeon has the option to safely intervene, these robotic systems shall be able to independently handle all environmental and adverse conditions without requesting or needing surgeon intervention.

The surgeon may supervise the procedure at any level.

### Supplementary information

Supplementary Information

Supplementary Data 1

## Data Availability

The datasets used and/or analyzed during the current study are available from the corresponding author on reasonable request. All FDA 510(k) summary files used in this research are available at https://www.accessdata.fda.gov/scripts/cdrh/cfdocs/cfpmn/pmn.cfm. All FDA De Novo summary files used in this research are available at https://www.accessdata.fda.gov/scripts/cdrh/cfdocs/cfPMN/denovo.cfm. All AccessGUDID records used in this research are available at https://accessgudid.nlm.nih.gov/.
