# Supplementary material for: Levels of autonomy in FDA-cleared surgical robots: a systematic review
Source: NPJ Digit Med. 2024 Apr 26;7:103. doi: 10.1038/s41746-024-01102-y (PMC11053143; doi:10.1038/s41746-024-01102-y)
Supplement: Supplementary file 1 — Supplementary Information [file 41746_2024_1102_MOESM1_ESM.pdf]

Supplementary Information

Levels of Autonomy in FDA-Cleared Surgical Robots:

A Systematic Review

Audrey Lee[1,2]; Turner S. Baker[1,2]; Joshua B. Bederson[1,2]; Benjamin I. Rapoport[1,2,*]

[1]Department of Neurosurgery, Icahn School of Medicine at Mount Sinai, New York, New York

[2]Sinai BioDesign, Icahn School of Medicine at Mount Sinai, New York, New York

*Corresponding Author:

Benjamin I. Rapoport, MD, PhD
Assistant Professor of Neurosurgery
Scientific Director, Sinai BioDesign Program
Icahn Medical School, Mount Sinai Medical System
Office: (212) 241-2763 / Cell: (646) 457-9250
Email: benjamin.rapoport@mountsinai.org

Document Contents:

Supplementary Table 1. Relevant definitions for Surgical Robots

Supplementary Methods. Data items

Supplementary References.

Separate File:

Supplementary Data 1. All surgical robots and their data collected in this study.

Supplementary Table 1. Definitions and existing taxonomic tools for Surgical Robots

Organization and document	Definition and comments	Reference
International Organization for Standardization, ISO 8373:2021 Robotics	Defines robot as a "programmed actuated mechanism with a degree of autonomy to perform locomotion, manipulation or positioning."<br>Defines autonomy as "ability to perform intended tasks based on current state and sensing, without human intervention." The degree of autonomy for a particular application is evaluated according to the "quality of decision-making and independence from human," which is parameterized by metrics defined in IEC/TR 60601-4-1.	1,2
International Organization for Standardization and International Electrotechnical Commission, IEC/TR 60601-4-1:2017 Medical electrical equipment — Part 4-1: Guidance and interpretation — Medical electrical equipment and medical electrical systems employing a degree of autonomy	Defines "Medical electrical equipment" as "electrical equipment having an applied part or transferring energy to or from the patient or detecting such energy transfer to or from the patient and which is: provided with not more than one connection to a particular supply mains; and intended by its manufacturer to be used in the diagnosis, treatment, or monitoring of a patient; or for compensation or alleviation of disease, injury or disability." This "includes those accessories as defined by the manufacturer that are necessary to enable the normal use of the medical electrical equipment."<br><br>Defines a Degrees of Autonomy taxonomy based on the ability of a medical equipment or system to independently generate possible options to achieve predefined goals, select an option, execute the selected option, and monitor data regarding the option execution.<br><br>This standard focuses only on the technical metrics of the system and fails to discuss practical developmental benchmarks and human-robot interactions, all of which are critical to ensure patient and surgeon safety throughout the advancement of the field.	2-4
International Organization for Standardization and International Electrotechnical Commission, IEC/CD 80601-2-77:2019 Medical electrical equipment — Part 2-77: Particular requirements for the basic safety and essential performance of robotically assisted surgical equipment	Defines "Robotically Assisted Surgical Equipment/System" as a "Medical Electrical Equipment/System that incorporates a Programmable Electrical Medical System actuated mechanism intended to facilitate the placement or manipulation of Robotic Surgical Instrument."<br>Defines "Robotic Surgical Instrument" as an "Invasive device with applied part, intended to be manipulated by Robotically Assisted Surgical Equipment/System to perform tasks in surgery".<br><br>Based on the definition of an invasive robotic surgical instrument, several robotic systems are outside the scope of this standard. For instance, robotic radiotherapy systems and robotized surgical microscopes are excluded. Other robotic systems are considered conditionally in scope, depending on if the instrument or part they are paired with could enter the surgical field and contact the patient.	3,5
US Food and Drug Administration	States that, by definition, there are no "surgical robots" on the market that perform minimally invasive surgical tasks autonomously. Instead, these systems are "robotically-assisted surgical devices" which perform tasks guided by the surgeon's direct control.<br>Ultimately, fully autonomous robotic surgical technology may be developed for untold uses.	6-8
Society of American Gastrointestinal and Endoscopic Surgeons and the Minimally Invasive Robotic Association, Consensus Document on Robotic Surgery	States that "robotic surgery" is an imprecise term widely used by the medical and lay press to refer to surgical technology that places a computer-assisted electromechanical device in the path between the surgeon and the patient. The more scientifically accurate term would be "remote tele-presence manipulators."<br>Defines robotic surgery as "a surgical procedure or technology that adds a computer technology enhanced device to the interaction between a surgeon and a patient during a surgical operation and assumes some degree of control heretofore completely reserved for the surgeon." Further clarifies that the definition "encompasses micromanipulators as well as remotely-controlled endoscopes in addition to console-manipulator devices. The key elements are enhancements of the surgeon's abilities, be they vision, tissue manipulation, or tissue sensing, and alteration of the traditional direct local contact between surgeon and patient."<br>States that "robots could use "artificial intelligence" to learn from the surgeon operating the device. Thus, robots could move from telemanipulators to skilled assistants in the future. If a robot acquired technical or cognitive knowledge from a large group of surgeons, it could ultimately serve as a computerized "colleague" to provide technical assistance in routine or unusual operative situations."	9
Attanasio et al. 2021<br>Haidegger et al. 2019<br>Fiorini et al. 2022<br>Yang et al. 2017<br>Fosch-Willaronga et al. 2023<br>Haidegger et al. 2022	The surgical robotics research community has proposed a more empirical scale of Levels of Autonomy for surgical robots, with Level 0–No autonomy, Level 1–Robot assistance, Level 2–Task-level autonomy, Level 3–Conditional autonomy, Level 4– High-level autonomy, and Level 5–Full autonomy. [10-14]<br><br>Although the consensus has been that most current surgical robots are at lower levels of autonomy, ambiguities and contradictions remain in how each level of autonomy is defined and how surgeons could intervene. For instance, some researchers defined that Level 0 surgical robots have no active robotic equipment, and procedures that use these systems are to be considered identical to non-robotic, fully manual cases. [11,13] Other researchers have defined that Level 0 surgical robots have no autonomy and exactly replicate surgeon movements, but at the same time may possess algorithmic autonomy to assist in tremor suppression and redundancy resolution of surgeon movements. [10,12] Some researchers also proposed that at higher levels of autonomy (Levels 4 and 5), surgeons are not required to supervise the procedure and may not even have the option to intervene. [11,13] Yet others have defined that constant supervision by the surgeon is needed even at these higher autonomy levels and thus the ability to intervene should still be required, such as through emergency stops or control handover to the surgeon. [4,10]	4,10-14

Supplementary Methods. Data items

We collected data on:

- The report:
    - 510(k) and De Novo databases: device classification name, device name, assigned 510(k) or De Novo number, summary or statement link, applicant or company name, applicant contact, correspondent or company name, correspondent contact, regulation number, classification product code, date received, final decision date, decision, regulation medical specialty, review panel, type of application, reviewed by third party (yes/no), combination product (yes/no), review link (if applicable), classification advisory committee, review advisory committee, reclassification order link (if applicable).
    - AccessGUDID database: Global Medical Device Nomenclature (GMDN) term(s), company name, brand name, primary record ID (public device record key), version or model, device ID, GMDN term code, GMDN term status, implantable (yes/no).

- The study/medical device: company name, device name, product website(s), key features (device description, technological characteristics), indications for use, approval pathway, first FDA clearance/grant date, assigned 510(k) or De Novo numbers, designated by FDA as AI/ML-enabled device (yes/no), marketed as AI/ML-enabled device (yes/no), performance data, older generation device name.

Supplementary References

1 ISO - International Organization for Standardization. in *Terms and definitions — General* Vol. ISO 8373:2021 (2021).

2 ISO - International Organization for Standardization. Vol. IEC 60601-4-1:2017 (2017).

3 Chinzei, K. Safety of Surgical Robots and IEC 80601-2-77: The First International Standard for Surgical Robots. *Acta Polytechnica Hungarica* (2019).

4 Fiorini, P., Goldberg, K. Y., Liu, Y. & Taylor, R. H. Concepts and Trends n Autonomy for Robot-Assisted Surgery. *Proc IEEE Inst Electr Electron Eng* **110**, 993-1011 (2022). https://doi.org:10.1109/JPROC.2022.3176828

5 ISO - International Organization for Standardization. Vol. IEC 80601-2-77:2019 (2019).

6 United States Food & Drug Administration. (ed United States Department of Health and Human Services) (2015).

7 United States Food & Drug Administration. (ed United States Department of Health and Human Services) (2020).

8 United States Food & Drug Administration. *Computer-Assisted Surgical Systems*, <https://www.fda.gov/medical-devices/surgery-devices/computer-assisted-surgical-systems> (2022).

9 Herron, D. M., Marohn, M. & The, S.-M. R. S. C. G. A consensus document on robotic surgery. *Surgical Endoscopy* **22**, 313-325 (2008). https://doi.org:10.1007/s00464-007-9727-5

10 Attanasio, A., Scaglioni, B., Momi, E. D., Fiorini, P. & Valdastri, P. Autonomy in Surgical Robotics. *Annual Review of Control, Robotics, and Autonomous Systems* **4**, 651-679 (2021). https://doi.org:10.1146/annurev-control-062420-090543

11 Haidegger, T. Autonomy for Surgical Robots: Concepts and Paradigms. *IEEE Transactions on Medical Robotics and Bionics* **1**, 65-76 (2019). https://doi.org:10.1109/TMRB.2019.2913282

12 Yang, G.-Z. *et al.* Medical robotics - Regulatory, ethical, and legal considerations for increasing levels of autonomy. *Science Robotics* **2**, eaam8638 (2017). https://doi.org:doi:10.1126/scirobotics.aam8638

13 Haidegger, T., Speidel, S., Stoyanov, D. & Satava, R. M. Robot-Assisted Minimally Invasive Surgery—Surgical Robotics in the Data Age. *Proceedings of the IEEE* **110**, 835-846 (2022). https://doi.org:10.1109/JPROC.2022.3180350

14 Fosch-Villaronga, E., Khanna, P., Drukarch, H. & Custers, B. The Role of Humans in Surgery Automation. *International Journal of Social Robotics* **15**, 563-580 (2023). https://doi.org:10.1007/s12369-022-00875-0
